# Longitudinal brain dynamics and prediction of craving changes in individuals with methamphetamine use disorder

**DOI:** 10.1038/s41398-026-04401-y

**Published:** 2026-09-02

**Authors:** Qing Song, Wenhan Yang, Zhe Du, Dongcheng Wang, Xinwen Wen, Jun Liu, Kai Yuan

**Affiliations:** 1 School of Life Science and Technology, Xidian University, Xi’an, Shaanxi 710126 China; 2 Department of Radiology, Second Xiangya Hospital, Central South University, Changsha, 410011 China; 3 Inner Mongolia Key Laboratory of Pattern Recognition and Intelligent Image Processing, School of Information Engineering, Inner Mongolia University of Science and Technology, Baotou, Inner Mongolia 014010 China; 4 Engineering Research Center of Molecular and Neuro Imaging Ministry of Education, Xi’an, Shaanxi China

**Keywords:** Neuroscience, Predictive markers

## Abstract

Individuals with methamphetamine use disorder (MUD) exhibited functional and structural brain recovery during abstinence. However, longitudinal neural changes of large-scale brain networks after prolonged abstinence remain unclear. Here, energy landscape analysis was applied to resting-state fMRI to characterize dominant activity patterns in 41 healthy controls and 40 individuals with MUD at baseline and after prolonged abstinence. We compared the dynamic characteristics of these patterns across groups and stages. Support vector regression model was constructed using baseline dynamic indices to predict craving changes after prolonged abstinence. We found that individuals with MUD exhibited rigid brain dynamics, which were correlated with abnormal brain network coordination and behaviors. Following prolonged abstinence, neural flexibility showed partial improvement, and dynamic indices could predict longitudinal craving changes. We revealed atypical brain network dynamics and their partial recovery during prolonged abstinence in MUD and suggested that dynamic indices could serve as biomarkers for predicting craving changes.

## Introduction

Methamphetamine (METH) is a toxic and addictive psychoactive substance that primarily affects the monoamine neurotransmitter system of the brain [1]. Methamphetamine use disorder (MUD) has become a global public health problem, causing severe health impairment and a widespread social burden [2]. Current pharmacotherapies show limited efficacy, and relapse rates remain high [3]. Therefore, elucidating the neurobiological mechanisms of MUD and identifying reliable biomarkers are essential for developing effective treatment strategies to reduce relapse risk.

Converging evidence suggested that individuals with MUD exhibited significant dysfunction and structural deficits in brain regions associated with reward processing, executive control, motivation, and decision-making [4–6]. Notably, longitudinal evidence has indicated that brain function and cognitive abilities in individuals with MUD can partially recover following prolonged abstinence, accompanied by a reduction in craving scores [7–9]. Most previous studies have concentrated on predefined regions of interest of brain networks or single behavioral measures, lacking a characterization of the longitudinal neural changes of brain networks. Previous research has shown that coordinated whole-brain neural dynamics serve as a critical foundation for flexible cognitive processes, enabling individuals to adapt to changing environmental demands and control excessive reward seeking [10–12]. However, the changes in whole-brain neural dynamics during prolonged abstinence and the relationship between brain dynamics and craving remain poorly understood.

Many methods have been used to investigate brain dynamics, including the sliding window approach and the hidden Markov model. However, the sliding window approach depends on an arbitrarily defined window length and primarily captures temporal fluctuations in pairwise functional connectivity [13]. The hidden Markov model mainly characterizes latent states and their transition probabilities, but provides relatively limited information regarding transition difficulty and the interpretability of state representations [14]. Recently, energy landscape analysis has provided a novel framework for characterizing the temporal changes in whole-brain neural activity patterns [15]. This approach uses the pairwise maximum entropy model (MEM) to construct an energy landscape and describes brain dynamics as the random movement of a ball across this landscape. In this framework, each local energy minimum corresponds to a stable spatial activity pattern representing a distinct brain state [16]. By quantifying the energy barriers between different local minima, this method can assess the difficulty of transitions between states, thus providing a more intuitive and interpretable characterization of brain activity patterns [17]. In this study, we conducted a longitudinal follow-up of individuals with MUD, applied energy landscape analysis to characterize longitudinal changes in brain network dynamics, and further explored the neural dynamics underlying craving.

As a core feature of addiction, craving has long been recognized as a significant risk factor for relapse [18]. During long-term abstinence, significant individual differences in craving changes were observed among individuals with MUD. Employing machine learning methods to identify underlying neurobiological markers associated with craving changes can aid in developing personalized treatment decisions and improving therapeutic outcomes [19]. Substantial evidence has indicated that various static brain biomarkers can predict changes in craving [20–22]. However, traditional static analyses require calculating functional connectivity over long periods, offering only static snapshots of the brain that fail to capture how the time-varying properties of large-scale brain functional networks give rise to individual variations in craving. Existing evidence suggested that individuals with high craving levels often exhibit prolonged durations of specific brain states and difficulty in switching between different network states [23]. Dynamic features can provide additional insights into temporal profiles, which may underlie the essential neural basis of individual differences in craving. Taken together, constructing a predictive model based on dynamic features has important implications for predicting craving changes.

The current study applied energy landscape analysis to characterize the large-scale brain network dynamics of 41 healthy controls (HC) and 40 individuals with MUD, and further investigated the across-network functional coordination underlying neural dynamics. Importantly, we tracked longitudinal alterations in brain dynamics following prolonged abstinence in individuals with MUD and examined whether baseline dynamic indices could predict longitudinal changes in craving. We hypothesized that individuals with MUD would exhibit aberrant whole-brain dynamics accompanied by increased functional segregation, with partial recovery of neural flexibility after long-term abstinence. Moreover, baseline dynamic indices could serve as potential biomarkers to predict longitudinal changes in craving.

## Methods

### Participants

This study included 41 HC and 40 individuals with MUD (Table 1). No formal a priori sample-size calculation was performed. The final sample comprised all eligible participants recruited during the study period who had usable MRI data. The individuals with MUD were assessed at baseline (MA-T1; abstinence ≤3.5 months) and re-assessed after sustained abstinence (MA-T2; abstinence ≥8 months). The inclusion criteria for methamphetamine abstainers were as follows: (1) ages 18–45 years and right-handed; (2) ability to understand and complete the questionnaire; (3) meeting the DSM-5 criteria for severe MUD (meets more than or equal to 6 criteria); (4) no history of neuropsychiatric disorders. Exclusion criteria for all subjects included: (1) a history of head injury; (2) the contraindications to MRI. All eligibility criteria and MRI quality-control thresholds were established before data analysis.

**Table 1:** Demographic and clinical characteristics of the participants.

	HC	MA-T1	Test	P
Age(years)	35.56 ± 10.18	32.05 ± 8.17	t = 1.701,df = 79	p = 0.0929
Sex(M/F)	34/7	26/14	χ² = 3.388	p = 0.0657
Education(years)	9.91 ± 2.57	9.85 ± 2.94	t = 0.1048,df =79	p = 0.9168
FTND	N/A	4.5 ± 2.27	N/A	N/A
AUDIT	N/A	5.65 ± 6.55	N/A	N/A
Age of first use(years)	N/A	26.78 ± 7.48	N/A	N/A
Duration of abstinenceat baseline(months)	N/A	3.43 ± 4.78	N/A	N/A
Craving	N/A	4.13 ± 2.71	N/A	N/A
BIS-11	N/A	83.56 ± 14.66	N/A	N/A
Dose of substance use(g)	N/A	1850.05 ± 2212.83	N/A	N/A
Duration of use(years)	N/A	8.09 ± 6.58	N/A	N/A

*HC* healthy controls, MA-T1 abstinence ≤3.5 months; MA-T2 abstinence ≥8months. *FTND* fagerstrom test for nicotine dependence, *AUDIT* alcohol use disorders identification test.

Nicotine dependence and alcohol use were evaluated using the Fagerström Test for Nicotine Dependence (FTND) and the Alcohol Use Disorders Identification Test (AUDIT), respectively. Methamphetamine craving scores were assessed using the visual analog scale (VAS; 0–10, where 0 indicates the weakest craving and 10 the strongest) to quantify changes in subjective craving intensity across the abstinence period. Among 40 individuals with MUD, 38 completed longitudinal VAS assessments at baseline (MA-T1, abstinence duration ≤ 3.5 months) and follow-up (MA-T2, abstinence duration ≥ 8 months) and were therefore included in the longitudinal craving-change and SVR analyses. The remaining two participants were excluded from these analyses because follow-up VAS data were unavailable. Impulsivity was assessed with the Barratt Impulsiveness Scale (BIS-11).

### Image acquisition and preprocessing

MRI scans were performed using a 3 T Siemens Skyra MRI scanner (Magnetom Skyra, Siemens, Germany) equipped with a 32-channel head coil. Sagittal BRAVO sequences were used to acquire 3D T1-weighted images with the following parameters: 176 sagittal slices, slice thickness = 1 mm, gap = 0 mm, field of view (FOV) = 256 mm × 256 mm, repetition time (TR) = 1450 ms, echo time (TE) = 2.03 ms, inversion time (TI) = 900 ms, flip angle = 30°, voxel size = 1 × 1 × 1 mm³. Resting-state fMRI data were acquired using a gradient echo echo-planar imaging (EPI) sequence with the following parameters: 36 axial slices, slice thickness = 4 mm, FOV = 220 × 220 mm, TR = 2000 ms, TE = 30 ms, flip angle = 80°, 225 volumes.

We preprocessed the resting-state data using the FMRIB Software Library (FSL, http://www.fmrib.ox.ac.uk/fsl/index.html) and Analysis of Functional NeuroImages (AFNI, http://afni.nimh.nih.gov/). First, to eliminate the impact of signal instability at the beginning of the scan, the first 10 time points were discarded. Slice timing correction was then applied to compensate for time shifts between slices during data acquisition. To reduce motion artifacts, head motion correction was performed, and data with translation exceeding 1.5 mm or rotation exceeding 1.5° were excluded to ensure signal quality. Subsequently, the structural images were corrected for tilting and underwent skull stripping, followed by registration with the functional images. The data were then standardized to the MNI152 template space. Based on this, spatial smoothing, intensity normalization, and noise removal were applied to improve the signal-to-noise ratio. Next, we performed peak removal on the time series to eliminate transient abnormal fluctuations and regressed out confounding signals during the scan to reduce non-neural noise. Finally, a band-pass filter with a frequency range of 0.01–0.08 Hz was applied to retain low-frequency oscillations and remove high-frequency noise and low-frequency drift, thereby extracting the relevant signal fluctuations associated with neural activity.

### Rois selection and functional networks segmentation

**Fig. 1:**
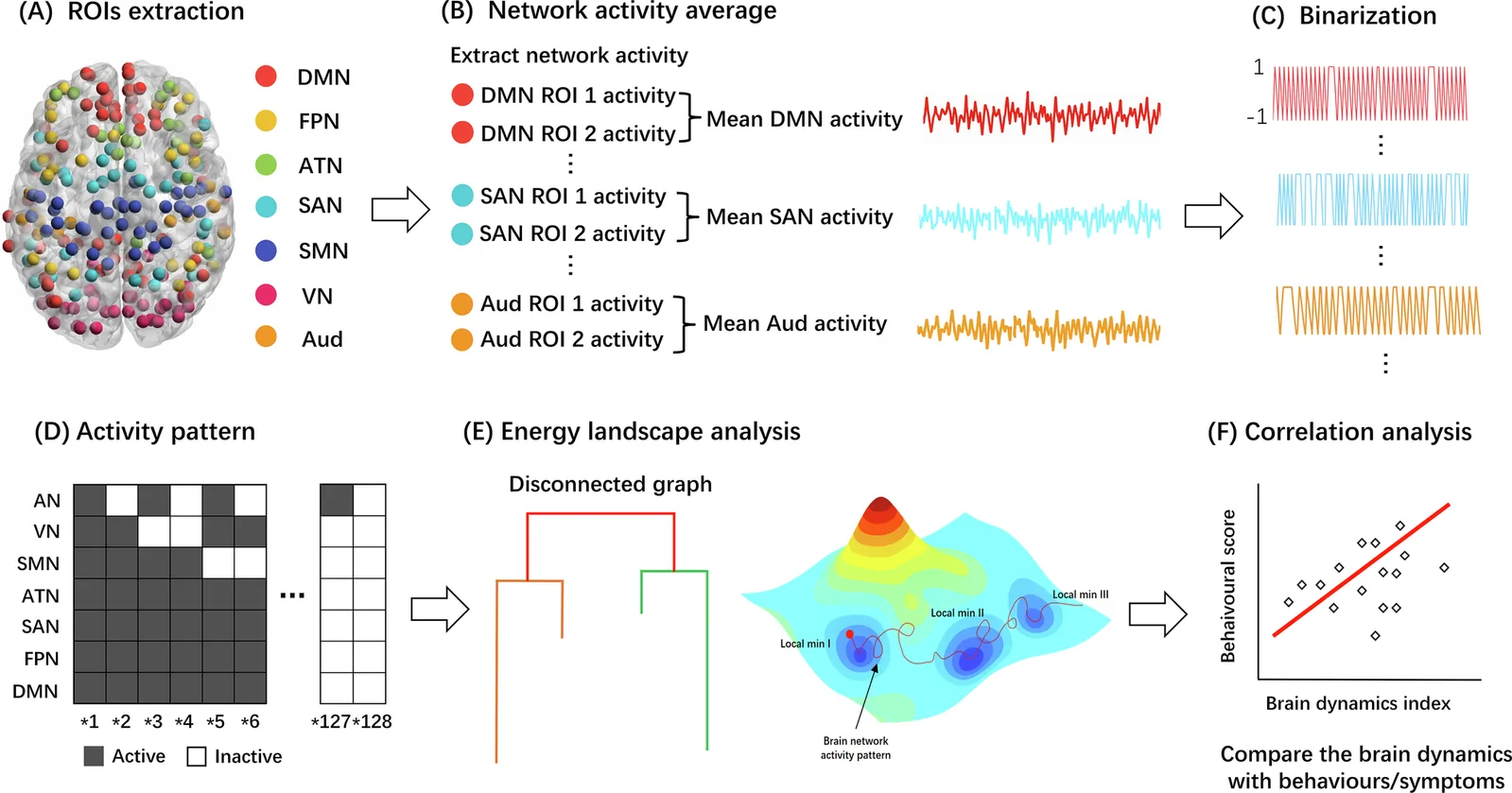
Flowchart of the energy landscape analysis. **A** A total of 214 regions of interest (ROIs) are extracted from the fMRI data and assigned to seven distinct functional networks. **B** The mean activity of each network is computed. **C** The continuous time series are binarized using the mean network activity as the threshold. **D** For N networks, a total of 2 ^N^ possible brain activity patterns can be defined. **E** Energy landscape analysis based on a pairwise maximum entropy model is performed to construct a disconnectivity graph and identify major brain states; brain dynamics are then characterized using random-walk simulations. **F** Finally, correlation analyses are conducted to examine the relationships between brain dynamical metrics and behavioral measures. DMN, default mode network; FPN, frontoparietal network; ATN, attention network; SAN, Salience Network; SMN, somatosensory and motor network; VN, visual network; Aud, auditory network.

Based on the previously published Power atlas [24], we extracted BOLD time series from 214 ROIs, each defined as a 3 mm radius sphere centered on the atlas coordinates (Fig. 1A). We then divided these ROIs into seven functionally distinct large-scale brain networks, including the default mode network (DMN), frontoparietal network (FPN), salience network (SAN), attention network (ATN), somatosensory and motor network (SMN), visual network (VN), and auditory network (Aud) [17]. To characterize brain network dynamics while reducing the influence of minor fluctuations that may reflect noise or local transient variability [25–27], we averaged the activity within each of the seven networks for each participant (Fig. 1B). Network activity was then binarized using the mean activity of each network as the threshold, with values above the mean indicating an active state (+1) and values below the mean indicating an inactive state (−1) (Fig. 1C). This mean-based thresholding approach avoids the use of an arbitrary absolute cutoff and provides a relatively balanced representation of active and inactive states for subsequent pairwise MEM analysis, which may reduce the risk of overfitting and improve the robustness of the results [15, 28, 29]. For each group, the binarized time series across participants were concatenated to generate sequences representing network activity over time.

### Fitting of the pairwise MEM

We used the Energy Landscape Analysis Toolkit in MATLAB to fit the pairwise MEM to the binarized time series of the seven networks in each group and construct the energy landscape of these networks [25, 27, 30, 31]. At any time t, the brain network activity pattern is ${V}^{t}=[{\sigma }_{1}^{t},\,{\sigma }_{2}^{t},...,\,{\sigma }_{N}^{t}]$, where ${\sigma }_{i}^{t}$ denotes the binarized activity of network i at time t with +1 indicating active and −1 indicating inactive. With N networks (N = 7), there are theoretically 2 ^N^ possible activity patterns (Fig. 1D).

In the maximum entropy model, when single network mean activity and pairwise interactions are constrained by the empirical data, the probability of each activity pattern V_k_ (k = 1,2,…,2 ^N^) follows a Boltzmann distribution: $P({V}_{k})={e}^{-E({V}_{k})}/{\sum }_{k=1}^{{2}^{N}}{e}^{-E({V}_{k})}$ with energy $E({V}_{k})=-{\sum }_{i=1}^{N}{h}_{i}{\sigma }_{i}({V}_{k})-\frac{1}{2}{\sum }_{i=1}^{N}{\sum }_{j=1,j\ne i}^{N}{J}_{{ij}}{\sigma }_{i}({V}_{k}){\sigma }_{j}({V}_{k})$. Here ${\sigma }_{i}({V}_{k})$ denotes the binarized activity of network i in pattern V_k_, h_i_ is the baseline activity of network i, and J_ij_ quantifies the pairwise coupling between networks i and j.

From P(V_k_) we further obtained the model based mean network activity ${ < {\sigma }_{i} > }_{m}\,={\sum }_{l=1}^{{2}^{N}}{\sigma }_{i}({V}_{l})P({V}_{l})$ and mean pairwise interaction ${ < {\sigma }_{i}{\sigma }_{j} > }_{m}\,=\,{\sum }_{l=1}^{{2}^{N}}{\sigma }_{i}({V}_{l}){\sigma }_{j}({V}_{l})P({V}_{l})$. We then used a gradient descent algorithm to iteratively adjust hi and J_ij_ so that ${ < {\sigma }_{i} > }_{m}$ and ${ < {\sigma }_{i}{\sigma }_{j} > }_{m}$ approached the empirical moments $< {\sigma }_{i} >$ and $< {\sigma }_{i}{\sigma }_{j} >$, thereby fitting the pairwise MEM parameters. Finally, we evaluated the accuracy of the pairwise MEM with the goodness of fit metric ${r}_{D}=\,\frac{({D}_{1}-{D}_{2})}{{D}_{1}}$, where D_k_ denotes the Kullback–Leibler (KL) divergence between the empirical distribution and the order k maximum entropy model, ${D}_{k}={\sum }_{l=1}^{{2}^{N}}{P}_{N}({V}_{l}){\log }_{2}\frac{{P}_{N}({V}_{l})}{{P}_{K}({V}_{l})}$, with p_N_ being the empirical distribution of brain-activity patterns.

### Identification of local minima

In energy landscape analysis, two brain activity patterns are considered adjacent if they differ only in the binarized state of a single network (+1 versus −1). We first identified all local energy minima as dominant brain states and then constructed a disconnectivity graph to depict the hierarchy among these stable states (Fig. 1E).

The procedure was as follows: (1) Each node corresponding to a pattern V_k_ has N neighboring nodes. (2) The initial energy threshold is set to the maximum energy among all 2 ^N^ nodes, denoted by E_th_. (3) Discard nodes with energy greater than E_th_, and remove all path connections incident to the discarded nodes. (4) E_th_ is then moved sequentially to the next highest energy value, and step (3) is repeated, producing progressively reduced networks in which local minima become isolated. (5) Finally, a disconnectivity graph is then constructed to visualize the local minima. Leaf nodes correspond to local minima, and internal nodes indicate branching points between stable states. This representation reveals the hierarchical organization and energy barriers among dominant brain states.

### Estimation of the sizes of dominant brain states

We quantified the dominance of each local minimum by the size of its basin, where a larger basin indicates a longer dwell time in the corresponding brain activity pattern [32]. Technically, we first selected a starting node i at random and then iterated over all 2 ^N^ nodes. For each node, if its energy exceeded that of any neighboring node, we moved it to a neighbor along the direction of decreasing energy. If its energy was not higher than all neighbors, the node was regarded as a local minimum and no further moves were applied. We repeated these steps until every state converged to a local minimum, and the starting node i was assigned as an element of the basin of the reached local minimum. Finally, we defined the basin size of a local minimum as the proportion of brain activity patterns in its basin relative to the total 2 ^N^ patterns.

### Random-walk simulation of dynamics for the energy landscape

We performed numerical simulations of the dynamic changes in brain activity patterns using the Metropolis–Hastings algorithm and the Markov chain Monte Carlo method [33–35]. Under this framework, any pattern V_i_ can transition to an adjacent pattern V_j_ only with probability ${P}_{{ij}}=\min [1,{e}^{E({V}_{i})-E({V}_{j})}]$. For each group, we executed 10^5^ repeated random walks starting from a randomly determined initial pattern [17]. We then summarized the trajectories of brain activity patterns as transitions between local minima and characterized brain dynamics by the transition frequencies and dwell times between those minima. To avoid the influence of initial conditions, we discarded the first 100 steps and estimated the dynamics from the stationary phase of the trajectories. Finally, we compared differences in brain dynamics between groups based on the random-walk simulations.

### Associations between brain dynamics and behaviors

Next, we examined associations between brain dynamics and behavior (Fig. 1F). We computed Pearson correlation coefficients between two behavioral measures (VAS craving scores and BIS-11 impulsivity scores) and dynamic indices in individuals with MUD.

### Functional coordination between brain networks

The dynamic changes in the brain on a short time scale are often influenced by more stable resting-state functional connectivity patterns over a longer duration [36, 37]. In the energy landscape model, each stable brain state exhibits a specific network activation pattern, which can further be divided into two distinct functional modules.

To quantify the degree of modular separation, we defined the strength of functional segregation as the difference between the average functional connectivity within modules and the average FC between modules. Higher isolation strength reflects more pronounced functional separation between networks, while lower values suggest enhanced coordination between networks. We then evaluated the correlation between these cross network functional integration and segregation patterns with brain dynamics metrics and behavioral scales.

### Prediction of craving changes based on brain dynamics indicators

Finally, we used support vector regression (SVR) to examine whether baseline brain dynamic indicators could predict longitudinal changes in craving. The input features consisted of 20 brain dynamic indicators derived from energy landscape analysis of empirical data and random-walk numerical simulations in 38 individuals from the MA-T1 group. These indicators included the occurrence frequency and duration of two major states and two minor states, as well as direct and indirect transition frequencies. The radial basis function (RBF) kernel was used in the SVR model. Model performance was evaluated using nested cross-validation, with leave-one-out cross-validation as the outer loop and 10-fold cross-validation within the training set as the inner loop. The inner loop was used to tune the hyperparameters of the RBF-kernel SVR model, including C, gamma, and epsilon. The searched parameter grid was C = [0.1, 1, 10], gamma = [0.01, 0.1, “scale”], and epsilon = [0.05, 0.1, 0.2]. The most frequently selected hyperparameter combination across the outer folds was C = 0.1, gamma = 0.01, and epsilon = 0.2. To evaluate the statistical significance of the predictions, we calculated the Pearson correlation coefficient between the observed craving change values and the model-predicted values. Additionally, we performed 1000 permutation tests by randomly shuffling the observed craving change values and repeating the complete prediction procedure to generate a null distribution, in order to test whether the model performance was significantly higher than chance.

### Statistical tests

The Shapiro-Wilk test was used to assess the normality of demographic and behavioral scale data. Homogeneity of variance was assessed using Levene’s test. Chi-square tests, two sample t-tests, Welch’s t-tests, or Mann-Whitney U tests were employed to examine group differences in demographics and behavioral scales. For the cross-sectional comparisons between the HC and MA-T1 groups, all brain dynamic metrics and the static functional segregation strength were compared using analysis of covariance (ANCOVA), with age, sex, and education as covariates. To examine longitudinal changes between MA-T1 and MA-T2, repeated measure analysis of covariance (RM-ANCOVA) was conducted separately for each brain dynamic metric and the static functional segregation strength, with time as the repeated-measures factor and baseline age, sex, education, FTND score, and AUDIT score as covariates. The statistical significance level was set at p < 0.05. Unless otherwise specified, all statistical tests were two-sided. The Benjamini-Hochberg false discovery rate (FDR) correction was applied separately to results derived from the empirical data and the random-walk numerical simulations, and separately for each group comparison (HC vs. MA-T1 and MA-T1 vs. MA-T2). For the empirical data, FDR correction was applied to appearance frequency, duration, direct and indirect transition frequency, and functional segregation strength. In the random-walk numerical simulations, FDR correction was performed for appearance frequency, duration, and direct and indirect transition frequency.

## Results

### Accuracy of model fitting

**Fig. 2:**
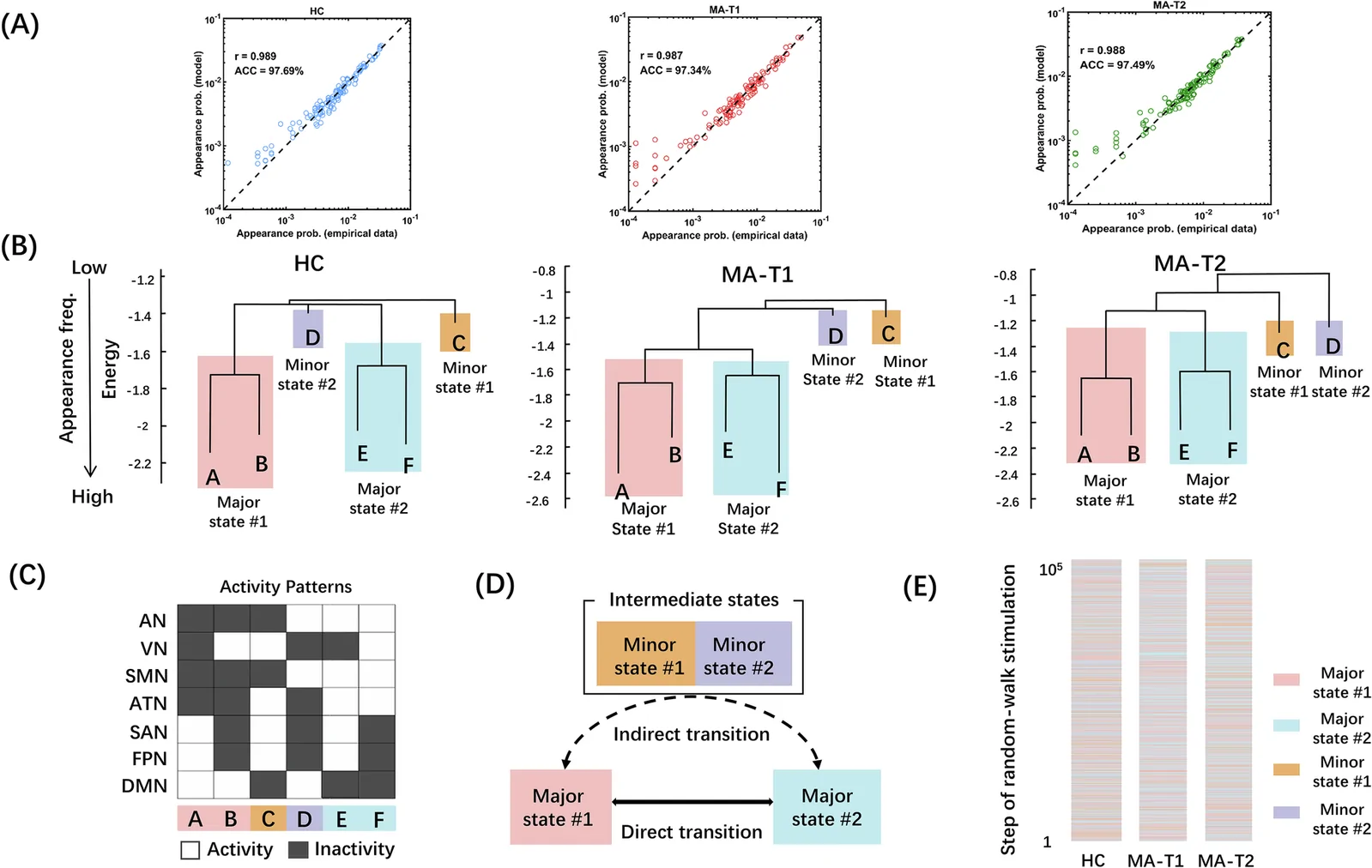
Pairwise maximum entropy model fitting and inferred energy landscapes. **A** The pairwise MEM accurately reproduced empirical pattern probabilities across all groups. **B** All groups showed similar hierarchical energy landscapes, each containing six local minima grouped into two major and two minor states. **C** The energy landscape analysis revealed that each local minimum corresponded to an identical large-scale brain network activity pattern across HC, MA-T1, and MA-T2. **D** Transitions between brain states were classified into two types of trajectories: direct transitions between the two major states and indirect transitions that proceeded via intermediate states. **E** Brain dynamics were further examined using 10⁵ random-walk simulation steps per group. The panel shows the occupancy distribution of brain states during the simulated trajectories, with different colors representing four distinct states. ACC, accuracy; HC, healthy controls; MA-T1, abstinence duration ≤ 3.5 months; MA-T2, abstinence duration ≥ 8 months; DMN, default mode network; FPN, frontoparietal network; ATN, attention network; SAN, Salience Network; SMN, somatosensory and motor network; VN, visual network; Aud, auditory network.

To investigate the brain dynamics characteristics of individuals with MUD and large-scale whole brain changes after long-term abstinence, we fitted the pairwise MEM to the binary network activity patterns. The model captured the distribution of brain states well in all groups, with stable performance, as the Pearson correlation coefficients between simulated and empirical occurrence probabilities exceeded 0.98 and the accuracy exceeded 0.97 (Fig. 2A).

### Identification of dominant brain states

The energy landscapes of HC, MA-T1, and MA-T2 exhibited a consistent hierarchical organization (Fig. 2B). Each landscape comprised six locally stable brain states, denoted as local minima A through F. Local minima A and B were located on one branch, while C and D were located on another, and their energies were lower than those of E and F, indicating greater stability and dominance. Accordingly, we defined A and B as major state #1, E and F as major state #2, and C and D as minor state #1 and minor state #2, respectively. Figure 2C presents the activation patterns of the seven networks for each local minimum. Major state #1 involved activation of the DMN and deactivation of the Aud, SMN, and ATN, whereas major state #2 involved coactivation of the Aud, SMN, and ATN with deactivation of the DMN. The two minor states likewise displayed opposite network activation patterns.

### Sizes of brain states

**Fig. 3:**
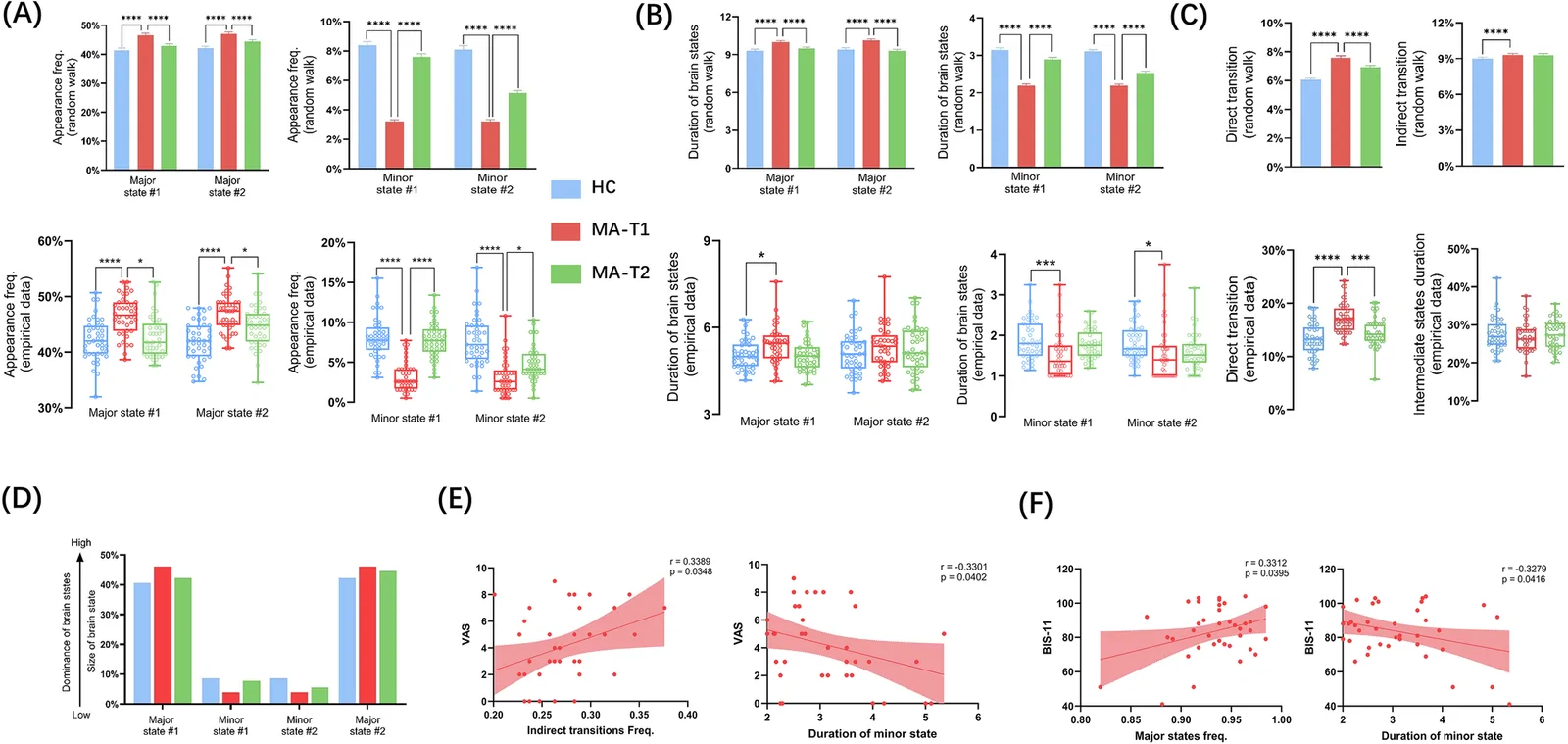
Results of the energy landscape analysis. **A** In both the random walk simulations and the empirical data, the MA-T1 group showed higher appearance frequency of major states and lower appearance frequency of minor states than the HC and MA-T2 groups. **B** In the simulations, the duration of major states increased while the duration of minor states decreased in the MA-T1 group. Consistently, the empirical data revealed a higher frequency of direct transitions in the MA-T1 group, mirroring the simulation results. **C** In the simulations, the frequency of indirect transitions was higher in the MA-T1 group than in the HC group, whereas other comparisons were not significant. **D** The degree of dominance of both the major states and the minor states differed among HC, MA-T1, and MA-T2. **E** In the MA-T1 group, visual analog scale scores were associated with the frequency of indirect transitions and the duration of minor states. **F** In the MA-T1 group, BIS-11 scores were associated with the appearance frequency of major states and the duration of minor states. *p < 0.05, **p < 0.01, ***p < 0.005, ****p < 0.0001; error bars indicate the standard deviation. HC Healthy control; MA-T1 abstinence ≤3.5 months; MA-T2 abstinence ≥8months.

We quantified the dominance of different brain activity patterns within the overall energy structure by calculating the proportion of area occupied by each local minimum basin within the energy landscape (Fig. 3D). Results revealed that in the MA-T1 group, major states occupied most of the entire energy terrain, accounting for approximately 92.0% of the total area (major state #1: 46.0%, major state #2: 46.0%). In contrast, the proportion of major states in the HC and MA-T2 groups was slightly lower, at 83.0% (major state #1: 41.0%, major state #2: 42.0%) and 87.0% (major state #1: 42.0%, major state #2: 45.0%), respectively. Concurrently, minor states in the MA-T1 group accounted for only approximately 8.0% of the energy landscape (minor state #1: 4.0%, minor state #2: 4.0%), while the proportion of minor states increased in the HC and MA-T2 groups to 17.0% (minor state #1: 8.5%, minor state #2: 8.5%) and 13.0% (minor state #1: 8.0%, minor state #2: 5.0%), respectively. No statistically significant differences were observed in the relative magnitudes of brain states across groups (chi = 5.52, P = 0.4796 in a Chi-square test).

### Characterization of brain dynamics indicators

After constructing the energy landscapes for HC, MA-T1, and MA-T2, we performed 10^5^ step random walk numerical simulations to obtain more realistic brain activity patterns (Fig. 2D). To better characterize brain dynamics, transitions between the two major states were classified into two types: the direct transition that does not pass through any other state and the indirect transitions that pass through intermediate states (Fig. 2E).

First, we calculated the appearance frequency of brain states on the energy landscape (Fig. 3A). In the empirical fMRI data, the two major states in the MA-T1 group appeared significantly more often than in the HC group (major state #1: F_1,76_ = 30.21, P_FDR_ < 0.001; major state #2: F_1,76_ = 38.45, P_FDR_ < 0.001), whereas the appearance frequency of the minor states was significantly reduced (minor state #1: F_1,76_ = 85.27, P_FDR_ < 0.001; minor state #2: F_1,76_ = 61.51, P_FDR_ < 0.001). These results were confirmed by the random walk simulations. Longitudinal analyses of MA-T1 and MA-T2 showed a decreasing trend in the appearance of major states (major state #1: F_1,34_ = 9.84, P_FDR_ = 0.011, η^2^ = 0.22; major state #2: F_1,34_ = 10.06, P_FDR_ = 0.011, η^2^ = 0.23) and an increasing trend in the appearance of minor states (minor state #1: F_1,34_ = 85.77, P_FDR_ < 0.001, η^2^ = 0.72; minor state #2: F_1,34_ = 6.53, P_FDR_ = 0.028, η^2^ = 0.16). Consistent with this partial recovery of neural dynamics, the random walk simulations also showed that, relative to MA-T1, the two major states appeared less frequently in MA-T2, whereas the two minor states appeared more frequently.

Figure 3B presents the results for the duration of the four brain states. In the random walk simulations, significant differences in duration were observed among HC, MA-T1, and MA-T2 for each brain state. However, in the empirical fMRI data, compared with HC, the MA-T1 group exhibited a longer duration in major state #1 (F_1,76_ = 5.65, P_FDR_ = 0.0275) and significantly shorter durations in the two minor states (minor state #1: F_1,76_ = 11.76, P_FDR_ = 0.0018; minor state #2: F_1,76_ = 6.23, P_FDR_ = 0.0232). For the longitudinal comparison, no significant changes in state duration were observed between MA-T1 and MA-T2.

Finally, we examined group differences in the frequency of direct transition and indirect transition between the two major states (Fig. 3C). In individual empirical data, direct transition was more frequent in MA-T1 than in the other two groups (MA-T1 vs. HC, F_1,76_ = 34.78, P_FDR_ < 0.001; MA-T1 vs. MA-T2, F_1,34_ = 16.26, P_FDR_ = 0.002, η^2^ = 0.32), and transition frequencies computed from the empirical fMRI time series showed similar results. However, for indirect transitions, only the random walk simulations showed a significantly higher frequency in MA-T1 than in HC (F_1,76_ = 112.56, P_FDR_ < 0.001), whereas other between-group comparisons were not significant.

### Association between brain dynamics indicators and behavioral scales

We observed abnormal whole-brain neural dynamics in individuals with MUD relative to HC based on the energy landscape analysis. To further examine how these abnormalities relate to behavior, we examined the associations between behavioral scale scores and key brain dynamics metrics, including frequency of appearance, frequency of direct and indirect transitions, and duration. In the MA-T1 group, craving scores were significantly positively correlated with the frequency of indirect transitions (r = 0.3389, P = 0.0348, Fig. 3E) and significantly negatively correlated with the duration of minor states (r = −0.3301, P = 0.0402, Fig. 3E). In addition, higher impulsivity was associated with a higher appearance frequency of major states (r = 0.3312, P = 0.0395, Fig. 3F) and a shorter duration of minor states (r = −0.3279, P = 0.0416, Fig. 3F). These findings suggest that the abnormal neural dynamics in individuals with MUD may be closely linked to impairments in craving regulation and impulse control.

### Association between across-network functional segregation strength and brain dynamics metrics and behavioral scales

Energy landscape analysis revealed stability and transition characteristics between various brain states, reflecting its underlying neurodynamic mechanisms. Building on this, we studied the structural features of neural dynamics at the functional network module level, hypothesizing that the abnormal brain dynamics are related to the functional segregation strength across-network modules.

**Fig. 4:**
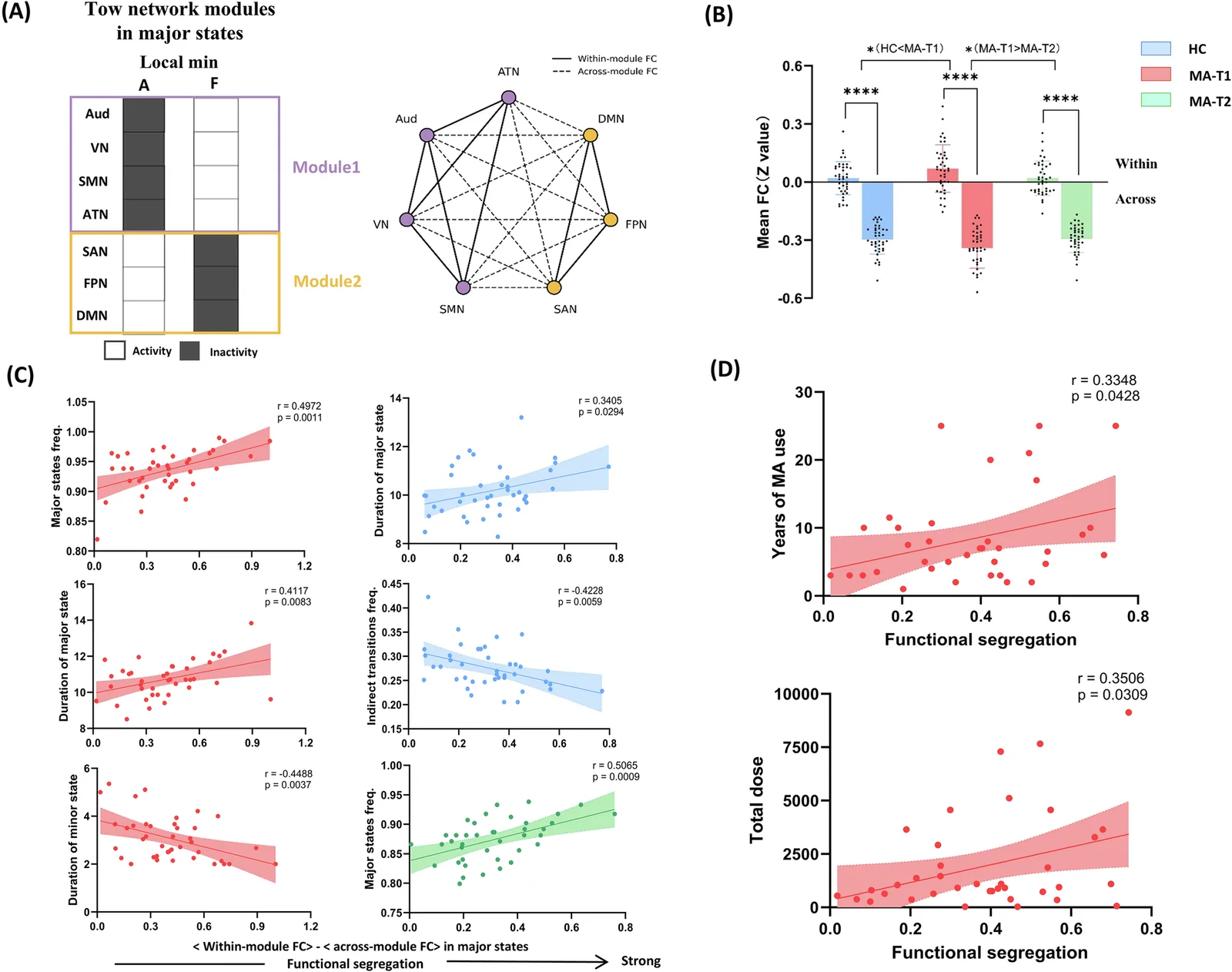
Functional segregation of large scale brain networks in the major states. **A** In local minima A and F of the major states, two complementary and anticorrelated modules are observed (Aud/VN/SMN/ATN module and SAN/FPN/DMN module). The strength of functional segregation between these two modules is defined as the difference between within module FC and between module FC. **B** All groups exhibited significant functional segregation, with the MA T1 group showing markedly stronger across-network functional segregation. **C** Associations between the strength of functional segregation and brain dynamical properties in each group. **D** Associations between abnormal functional segregation and behavioral measures in the MA T1 group. HC, healthy controls; MA-T1, abstinence duration ≤ 3.5 months; MA-T2, abstinence duration ≥ 8 months; DMN, default mode network; FPN, frontoparietal network; ATN, attention network; SAN, Salience Network; SMN, somatosensory and motor network; VN, visual network; Aud, auditory network.

As shown in Fig. 4A, the two major states, local minima A and F, exhibited complementary activity patterns, which were divided into two modules based on the activation of each network: the Aud/VN/SMN/ATN module (module 1; VN) and the SAN/FPN/DMN module (module 2; SAN; FPN). Significant functional segregation strength was observed in HC, MA-T1, and MA-T2 (HC: t_40_ = 12.81, MA-T1: t_39_ = 11.60, MA-T2: t_39_ = 12.67, P < 0.001 in a one-sample t-test, Fig. 4B). In the MA-T1 group, within-module and across-module FC were significantly higher than those in the HC and MA-T2 groups (MA-T1 vs HC: F_1,76_ = 4.83, P_FDR_ = 0.0379; MA-T1 vs. MA-T2: F_1,34_ = 7.22, P_FDR_ = 0.0244, η^2^ = 0.18, Fig. 4B).

We further investigated the relationship between across-network functional segregation strength and brain dynamics metrics as well as behavioral scale scores. In the MA-T1 group, this abnormal across-network functional segregation was significantly positively correlated with the appearance frequency (r = 0.4972, P = 0.0011, Fig. 4C) and duration (r = 0.4117, P = 0.0083, Fig. 4C) of the major states, and negatively correlated with the duration of the minor states (r = −0.4488, P = 0.0037, Fig. 4C). Similarly, in the MA-T2 group, the appearance frequency of the major states was positively correlated with the functional segregation strength (r = 0.5065, P = 0.0009, Fig. 4C). In the HC group, functional segregation was significantly positively correlated with the duration of the major states (r = 0.3405, P = 0.0294, Fig. 4C) but negatively correlated with the indirect transition frequency (r = −0.4228, P = 0.0059, Fig. 4C). Additionally, in the MA-T1 group, the strength of functional segregation was significantly positively correlated with both the years of MA use (r = 0.3348, P = 0.0428, Fig. 4D) and the total dose (r = 0.3506, P = 0.0309, Fig. 4D).

### Predicting longitudinal changes in craving based on brain dynamics characteristics

**Fig. 5:**
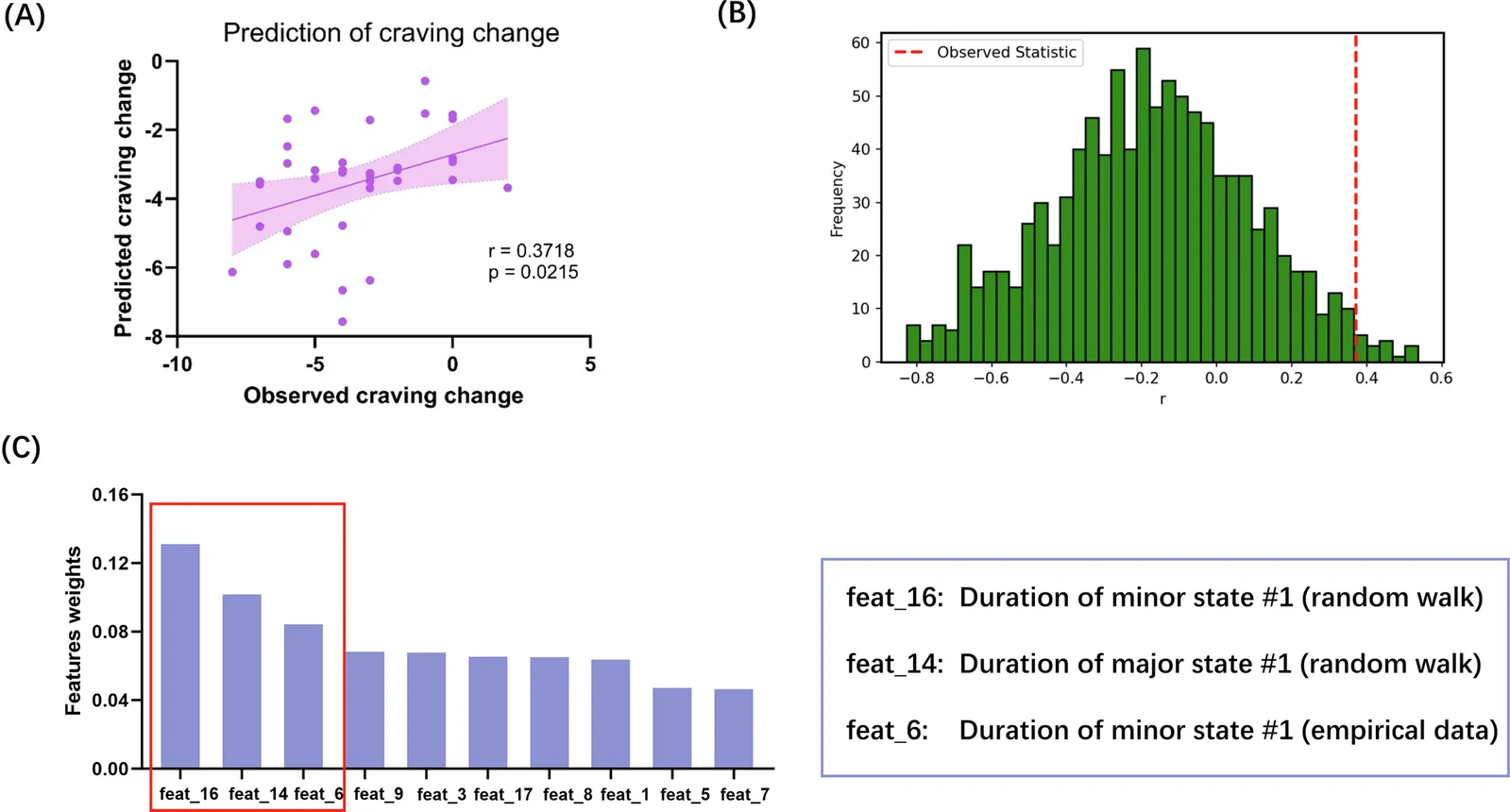
Prediction of long-term craving change using brain dynamical metrics. **A** Correlation between the observed change in craving and the change predicted by the SVR model. **B** Results of 1000 permutation tests for the correlation coefficient. **C** Feature weights of each brain dynamical metric in the craving prediction model and the top three ranked features. feat_9: Indirect transition (random walk); feat_3: Appearance frequency of minor state #2 (random walk); feat_17: Duration of minor state #2 (empirical data); feat_8: Direct transition (random walk); feat_1: Appearance frequency of major state #2 (random walk); feat_5: Duration of major state #2 (random walk); feat_7: Duration of minor #2 (random walk).

Finally, we utilized the Support Vector Regression (SVR) model with the brain dynamics features of 38 participants from the MA-T1 group as input variables to successfully predict individual longitudinal craving changes. The predicted and observed craving change scores were significantly positively correlated (r = 0.3718, P = 0.0215; one-sided permutation P < 0.05; Fig. 5A, B). To further identify the key contributing features of the model, we computed the top 10 variables based on their predictive contribution, with the top three dynamic features being the duration of simulated minor state #1, the duration of simulated major state #1, and the duration of actual minor state #1 (Fig. 5C). These results indicate that the brain stay patterns across different states have significant predictive value for individual craving changes, revealing a potential link between dynamic features and behavioral changes.

## Discussion

In the current study, we employed energy landscape analysis to characterize large-scale functional network dynamics in MUD and to examine changes of brain dynamics following prolonged abstinence. Energy landscape analysis offers an advantage over other approaches for characterizing brain dynamics because it captures both the stability of brain states and the difficulty of transitions between them, thereby providing a more intuitive and interpretable framework for understanding brain function. Applying this framework, we found that individuals with MUD exhibited rigid brain dynamics and increased functional segregation strength that were significantly correlated with behavioral measures. Furthermore, the dynamic indices in individuals with MUD were partially recovered following prolonged abstinence, accompanied by improved neural flexibility. Finally, baseline dynamic indices could predict craving changes during prolonged abstinence, particularly the duration of major state #1 and minor state #1. In conclusion, these findings provided novel insights into the longitudinal recovery trajectories of brain network dynamics in individuals with MUD and highlighted the potential of brain dynamic metrics as biomarkers for predicting craving changes.

The brain maintains dynamic equilibrium by transitioning between multiple stable brain states, thereby adapting to the constantly changing environmental conditions [38]. Our study revealed that individuals with MUD exhibited markedly rigid neural dynamics. This rigidity was characterized by increased occurrence and duration of the two major brain states, and direct transitions between these states occurred with abnormally high frequency. Previous studies have shown that individuals with substance use disorders exhibited imbalanced brain state dynamics, accompanied by altered patterns of brain state transitions [39]. Dynamic transitions between brain states were not the result of the activity of isolated neurons or single brain regions, but rather the process of functional networks dynamically configuring over time under global topological constraints [40]. Our results revealed that local minima A and F exhibited complementary brain activation patterns across two distinct network modules (Aud/VN/SMN/ATN module and SAN/FPN/DMN module), and individuals with MUD showed significantly higher functional segregation than HC. A previous study observed increased functional connectivity between the FPN and DMN, as well as between the SAN and DMN in individuals with MUD [41]. In addition, the functional connectivity between the left dorsolateral prefrontal cortex (FPN) and the left middle occipital gyrus (VN) showed a significant decrease [42]. Together with these findings, we revealed the increased across-network module segregation in individuals with MUD. Neural activity on short time scales is constrained by long-term static functional connectivity [36, 43]. Therefore, we further investigated the across-network functional coordination underlying neural dynamics. Our results showed that aberrant segregation between two network modules in individuals with MUD was associated with abnormally stable major brain states and prolonged duration. This aberrant across-network functional segregation may underlie the abnormally stable brain dynamics in individuals with MUD [11]. Typically, the brain maintains a relatively stable global network architecture while flexibly modulating local network parameters in response to specific cognitive demands, thereby facilitating communication between different large-scale functional networks [44, 45]. Our results suggested that METH abuse may impair the dynamic properties of brain networks, thereby compromising effective communication. Notably, we observed that abnormal neural dynamics in the MA-T1 group were associated with higher craving and impulsivity, while aberrant functional segregation was linked to duration of substance use and drug dosage. A recent study revealed that individuals with MUD exhibited decreased global topological properties, and the reduction of small-world properties was significantly associated with impulsivity and craving [46]. Additionally, the duration of functional network states was associated with the total METH usage [47]. In summary, our findings demonstrated the aberrant neural dynamics and disrupted brain network coordination in individuals with MUD, providing new evidence for the neurobiological mechanisms of methamphetamine addiction.

Individuals with MUD showed serious whole-brain dynamics perturbation, manifesting as an inability to flexibly switch between distinct network activation patterns. However, these rigid brain dynamics were significantly alleviated following prolonged abstinence. Specifically, the occurrence and duration of major states decreased, those of minor states increased, and the frequency of direct transitions between the two major states was reduced. These findings suggested that the brain was no longer rigidly confined to a few dominant states. Additionally, we observed a decrease in functional segregation strength in the MA-T2 group, indicating enhanced across-module connectivity. Increasing evidence suggested that individuals with MUD exhibited varying degrees of functional and structural brain recovery during abstinence [48–50]. However, most studies have primarily focused on changes in static brain properties, and the phenomenon of brain recovery within large-scale brain networks following prolonged abstinence has yet to be fully elucidated. The functional brain networks can dynamically switch between segregated and integrated states. Notably, the integrated state relies on flexible transitions between distinct brain states and serves as a critical foundation for efficient cognitive processing [40, 51]. Previous study has found that physical exercise can reshape dynamic functional reorganization and restore brain functional flexibility in individuals with MUD, highlighting the potential for functional recovery following long-term impairment [52]. Furthermore, the occurrence of brain network states characterized by a homeostatic balance between integration and segregation exhibited a significant positive correlation with the duration of abstinence in individuals with MUD [47]. Taken together, our results revealed the dynamic reconfiguration of network modules and the gradual recovery of the neural dynamics in individuals with MUD after prolonged abstinence.

Finally, the predictive model based on dynamic indices could predict the craving changes of individuals with MUD after prolonged abstinence. Notably, the duration of major state #1 and minor state #1 showed the most significant contributions to the prediction. Based on connectome-based predictive modeling, the DMN and SAN were identified as critical large-scale networks for craving prediction [53]. The DMN functions were related to self-referential processing, introspection, and value evaluation [54]. Individuals with SUD exhibited significantly increased DMN activity during abstinence, and this neural alteration was thought to be associated with increased craving scores [55–57]. The SAN played a key role in salience processing and interoceptive awareness, and facilitated state switching between the DMN and the executive control network [58]. In this study, the MA-T1 group exhibited prolonged dwell times in major state #1 dominated by DMN activation, indicating overactivity of DMN. Conversely, the reduced duration of minor state #1 characterized by SAN activation reflected impaired functional engagement. Prior work has shown that increased time spent in the DMN state and decreased time spent in the SAN state were associated with heightened activation in the anterior insula and dorsal anterior cingulate cortex during cue-reactivity tasks, predicting stronger cue-induced craving [59]. These findings revealed that brain dynamic indices contain craving-related information, providing new insight into predicting craving changes in individuals with MUD after prolonged abstinence.

Taken together, these findings may have potential clinical and translational implications. First, baseline brain dynamic indices may help identify individuals with MUD who are more likely to show poorer craving improvement during abstinence, thereby providing complementary information for individualized risk stratification and follow-up planning. Second, because these dynamic abnormalities showed partial recovery after prolonged abstinence and were associated with large-scale Fnetwork coordination, they may provide potential targets or objective outcome measures for future interventions aimed at improving network flexibility and craving regulation. However, these potential clinical and translational implications should be interpreted cautiously, and future studies with larger samples, independent cohorts, and more densely sampled longitudinal designs are needed to further validate their robustness and practical utility.

There are still several limitations in this study. Firstly, a relatively small sample size may limit the accuracy of statistical inference and the generalizability of conclusions. Future work should use larger samples and multicenter data to enhance robustness. Secondly, the design only included two longitudinal time points, limiting the ability to capture continuous neurodynamic changes throughout the entire abstinence process. Future research should extend the follow-up period to reveal the dynamic evolutionary trajectory of brain functional networks during different stages of abstinence. Thirdly, although this study focused on longitudinal changes in brain dynamics and craving in individuals with methamphetamine use disorder, potential comorbid factors were not assessed in sufficient detail, which may limit the specificity of the findings. Future studies should incorporate more comprehensive clinical assessments to further examine the effects of these comorbid factors during abstinence. Finally, although energy landscape analysis effectively characterizes stable brain state structures and transition patterns, this method is also sensitive to parameter selection. Future research should integrate more advanced modeling methods to improve the accuracy of interpreting brain dynamic processes.

## Conclusion

Using energy landscape analysis, we characterized longitudinal alterations in large-scale brain-network dynamics in individuals with MUD. Relative to healthy controls, MUD showed atypical neural dynamics and disrupted functional coordination that partially recovered following prolonged abstinence. Importantly, baseline dynamical features predicted longitudinal changes in craving, identifying candidate biomarkers. These findings elucidate mechanisms of brain dysfunction and recovery in MUD and may inform targeted clinical strategies for addiction.

## Data Availability

The data and materials that support the findings of this study are available from the corresponding author upon reasonable request.

## Code availability

The analysis code supporting the findings of this study is available from the corresponding author upon reasonable request.
